# Implementation and evaluation of the virtual Graded Repetitive Arm Supplementary Program (GRASP) for individuals with stroke during the COVID-19 pandemic and beyond

**DOI:** 10.1093/ptj/pzab083

**Published:** 2021-03-04

**Authors:** Chieh-ling Yang, Seonaid Waterson, Janice J Eng

**Affiliations:** Department of Physical Therapy, University of British Columbia, Vancouver, BC, Canada; Rehabilitation Research Program, Vancouver Coastal Health Research Institute, Vancouver, BC, Canada; Stroke Recovery Association of British Columbia, March of Dimes Canada, Vancouver, BC, Canada; Department of Physical Therapy, University of British Columbia, Vancouver, BC, Canada; Rehabilitation Research Program, Vancouver Coastal Health Research Institute, Vancouver, BC, Canada

**Keywords:** Arm, Hand, Exercise, Stroke, Implementation, Telerehabilitation, RE-AIM

## Abstract

**Objective:**

Given the uncertainty with the COVID-19 pandemic, implementing telerehabilitation that enables the remote delivery of rehabilitation services is needed to mitigate the spread of COVID-19. We studied the implementation and the effectiveness of the virtual Graded Repetitive Arm Supplementary (GRASP) Program delivered and evaluated via videoconferencing in individuals with stroke.

**Methods:**

The RE-AIM (Reach, Effectiveness, Adoption, Implementation, Maintenance) framework with mixed methods was used to evaluate the implementation of the two iterations of the program delivered by a nonprofit organization during the pandemic.

**Results:**

*REACH*: Seventeen people were screened, 13 people were eligible, and 11 consented to participate in the study. *EFFECTIVENESS*: Between baseline and post-test, participants with stroke demonstrated significant improvement in upper extremity function (Arm Capacity and Movement Test) and self-perceived UE function (Stroke Impact Scale). *ADOPTION*: Factors that facilitate program uptake by the staff were well-planned implementation, appropriate screening procedure, and helpful feedback from the audits. All staff felt comfortable using videoconferencing technology to deliver the program despite some technical difficulties. Factors contributing to ongoing participation included that the participants liked the group, they perceived improvements, and the instructor was encouraging. Only one participant with stroke was not comfortable using videoconferencing technology. *IMPLEMENTAION*: The program was implemented as intended as evaluated by a fidelity checklist. Participants’ adherence was high, as verified by the average attendance and practice time. *MAINTENANCE*: The organization continued to offer the program.

**Conclusion:**

The virtual GRASP program was successfully implemented. Although the program was effective in improving both measured and perceived UE function in a small sample of individuals with stroke, caution should be taken in generalizing the results.

**Impact:**

Implementing telerehabilitation is crucial to optimize patient outcomes and reduce the spread of COVID-19. Our findings provide guidance on the process of delivering a UE rehabilitation program remotely via videoconferencing for stroke. Moreover, insights that arise from this study also inform the implementation of other telerehabilitation services.

## [H1] Background

The WHO announced Coronavirus disease 2019 (COVID-19) as a pandemic in March 2020.[1] Based on some mathematical models, the overall duration of the COVID-19 pandemic could last into 2022, with the possibility of a resurgence in contagion as late as 2024.[2] As COVID-19 is transmitted from person-to-person, rehabilitation services that can be delivered remotely via communication technologies (ie, telerehabilitation)[3] without in-person contact are needed to mitigate the spread of COVID-19. While telerehabilitation has some key advantages such as eliminating the need for in-person contact and travel, and increased accessibility for people in rural and remote areas, the challenges associated with telerehabilitation have been well-documented.[4] Some common challenges included the inability to conduct hands-on treatment and the lack of expertise to troubleshoot the communication technology.[5,6] Thus, in times of COVID-19 and beyond, understanding how to implement and deliver a virtual rehabilitation exercise program is a matter of priority.

As approximately 88% of individuals with stroke did not regain complete functional recovery in their upper extremity (UE) at 6 months post-stroke,[7] stroke survivors often need continuous rehabilitation services to improve their UE function after being discharged home. The Graded Repetitive Arm Supplementary Program (GRASP) is a self-administered UE rehabilitation intervention designed for individuals with stroke based on intensive, repetitive, and task-specific practice.[8,9] It has been suggested by the Canadian Stroke Best Practice Recommendations for UE management.[10] Our recent study showed the effectiveness and successful implementation of the GRASP as a community program in a group setting (ie, GRASP community program) delivered by a local nonprofit organization (Stroke Recovery Association of British Columbia, an affiliate of the March of Dimes Canada, SRABC/MODC) at a local community center.[11] The GRASP community program consisted of ten weekly one-hour group classes and individualized homework exercises. The organization had delivered the in-person GRASP community program in a local community center since April 2019. However, due to the COVID-19 pandemic and related policies for social distancing, the organization decided to adapt the in-person GRASP community program into a virtual program (ie, virtual GRASP program) delivered via videoconferencing.

The purpose of this study was to evaluate the implementation process from the two iterations of the virtual GRASP program delivered via videoconferencing by a nonprofit organization using the RE-AIM framework (Reach, Effectiveness, Adoption, Implementation, and Maintenance). The findings of this study will not only inform the considerations of the successful implementation of the virtual GRASP program but also allow for insight into the delivery of other telerehabilitation programs during the COVID-19 pandemic and beyond.

**[H1] Methods**

**[H2]** *Implementation overview*

Two virtual GRASP programs were delivered from June to August in 2020. The two programs were delivered over the same period of 10 weeks but on different days in a week. Promotion and participant screening begun one month prior to the commencement of the program. To ensure the program fidelity, two audits were conducted by the research team member during in-class observation via videoconferencing in the 2$^{nd}$ and 6$^{th}$ weeks and the audit feedback was then provided to the instructor. The research team, as an outreach facilitator [12], provided support and consultation to assist transforming the in-person community program to a virtual format delivered by the SRABC/MODC. The SRABC/MODC regional coordinator provided operational and logistical oversight including in-kind promotion, administration, instructors, recruitment of volunteers, supervision of the instructor, and set cost-recovery fees to cover the salary of the instructor. The virtual GRASP program instructor screened potential participants, led the virtual program, and supervised and trained the volunteers. The virtual GRASP program volunteers assisted with classes under the supervision by the program instructor. The roles of the research team and SRABC/MODC were summarized in Supplemental Appendix 1.

**[H2]** *Virtual GRASP program*

The virtual GRASP program was adapted from the GRASP community program (http://neurorehab.med.ubc.ca/grasp/). Details regarding the virtual program are summarized using the Template for Intervention Description and Replication (TIDieR) checklist [3] in Table 1. The program consisted of 10 weekly one-hour group classes delivered via videoconferencing using Zoom software (Zoom Video Communications Inc., 2016) and individualized homework exercises. Participants were responsible for completing one hour of GRASP exercises daily and logging the practice time in the manual (Suppl. Appendix 2). If available, participants were encouraged to have their caregivers of family members attend the weekly class. The program fee was 85 CAD, reflecting the cost of staffing and manuals. The typical schedule of a weekly class started with 15 mins of experience sharing and discussion, followed by 30 mins of exercise progression and modification, and ended with 15 mins of goal setting and wrap-up (Suppl. Appendix 3). General principles of exercise progression and modification were outlined in the GRASP instructor manual (https://neurorehab.med.ubc.ca/grasp/grasp-manuals-and-resources/). One instructor led the weekly class, and two to three volunteers were available to assist with the classes. Before the program started, the participant manuals were mailed to the participants. The participants were responsible to source their own equipment for the program according to an equipment list provided by the SRABC/MODC. Most of the equipment was available in the home except for weights, therapy putty, and hand grippers. Information on where to purchase the equipment and alternatives (eg, playdough to substitute therapy putty) was provided to the participants. To ensure the safety of participants during online classes, the participants were asked to leave their cameras on so that the staff could visually monitor participants' conditions during online classes. The emergency contact information, home address, and phone number of each participant were on hand in case of an emergency or medical event. If the staff were concerned about the participant's safety, the staff would contact the person by phone and offer assistance by calling 911 or their emergency contact person. The instructor also reviewed the program safety guideline (i.e., follow the instructions provided by the instructor) with the participants during the screening process.

**[H2]** *Participants with stroke*

Individuals with stroke interested in attending the program contacted the SRABC/MODC coordinator and then were screened via videoconferencing by the program instructor. Eligibility criteria were as follows: (1) ≥ 19 years old, (2) diagnosis of stroke, (3) difficulty using the affected UE, (4) some voluntary movement in the affected UE, including the ability to lift the affected arm to the chest level and hold for 5 seconds, some ability to extend the affected wrist, and some ability to grasp and release an object such as a cup handle, 5) living in the community, 6) able to log into a videoconference to attend the weekly class, 7) able to understand and follow instructions, and 8) willing to practice GRASP exercises daily. Eligibility criterion #4 was assessed by asking the potential participants to lift the affected arm to the chest level (approximately 60-70 degrees of shoulder flexion), extend the affected wrist (visible active wrist extension from a flexion position), and to grasp and release an object (eg, a cup handle, a block, a pill bottle, etc.). Participants with severe pain that prevented the movement in the affected arm and hand were excluded. Eligible participants could register for the program. At that point, they were approached to participate and consent to an optional research study by the research team.

**[H2]** *Staff*

One paid instructor with experience leading the GRASP community program led the program and had received prior GRASP training through an in-person group workshop led by a physical therapist about a year prior. The instructor was a health science student. One volunteer was a registered kinesiologist, and the other two volunteers were health science students. Both the instructor and volunteers received training on aphasia provided by the organization. The aphasia training consisted of a webinar on aphasia knowledge provided by a speech-language pathologist and an online learning module on Supported Conversation for Adults with Aphasia (SCA™) from the Aphasia Institute (https://www.aphasia.ca/communityhub/).

**[H2]** *Data collection and analysis*

The implementation was examined using the RE-AIM framework (Reach, Effectiveness, Adoption, Implementation, and Maintenance), which provided a systematic and comprehensive framework to evaluate the translation of an evidence-based intervention to practice (http://www.re-aim.org/) [13]. The framework encompasses five elements to be assessed (Reach, Effectiveness, Adoption, Implementation, and Maintenance) and it has been widely used to design, implement, and evaluate research.[14] Table 2 provides an overview of the variables under each RE-AIM element, the data sources used to assess each variable, and the data collection timeline. All data collection was done virtually using videoconferencing software (Zoom Video Communications, Inc.). A newly developed online assessment, Arm Capacity and Movement test (ArmCAM)[15], was used to assess the body functions and activities according to the International Classification of Functioning, Disability and Health (ICF). The Rating of Everyday Arm-use in the Community and Home Scale (REACH) and the Stroke Impact Scale Hand subscore (SIS-Hand) were used to assess the Activities domain. The ArmCAM has good reliability and validity with the Fugl-Meyer Assessment for upper extremity, Action Research Arm Test, SIS-Hand, and REACH[15]. The ArmCAM consists of 10 items that measure gross movements (2 items), supported reach (1 item), functional reach and grasp movements (3 items), functional wrist movement (1 item), and fine motor skills (3 items). All items are rated on a 4-point ordinal scale (0, can perform no part of task; 1, perform task partially; 2, complete task, but slower, with difficulty, or using compensatory strategies; 3, perform task normally). Assessment objects include one magazine, one sheet of paper towel, two cups, one jar with a lid, one soup can, and three coins. It takes approximately 20 minutes to administer the test. Scored participant videos of the ArmCAM are available at the link: https://www.youtube.com/watch?v=CsplVu_U-mg.

Descriptive analysis was used to summarize baseline demographic, stroke characteristics, individual changes in clinical assessment scores, responses to the questionnaires, and program and individual records. Effectiveness shown by the differences across three time points (baseline, post-test, follow-up) was assessed by the Friedman tests with a within-group factor of time. Post-hoc paired comparisons (baseline vs. post-test; baseline vs. follow-up) were done using the

Wilcoxon signed-rank test. The effect size *r* (small effect: $0.1 < r < .3$; medium effect: $0.3 < r < .5$; large effect: $r \geq .5$) is calculated as Z statistic from the Wilcoxon signed-rank test divided by the square root of the sample size.[16] The significance level was set as $p < .05$. SPSS 25.0 software (IBM, Armonk, NY, USA) was used for statistical analyses.

**[H2]** *Role of the Funding Source*

The funders played no role in the design, conduct, or reporting of this study.

**[H1] Results**

**[H2]** *Reach – Size and representativeness of enrolled participants*

Seventeen individuals were screened for eligibility and 13 were eligible. Four did not meet the inclusion criteria due to insufficient voluntary movement in the affected arm/hand. Among the 13 eligible individuals, one person did not register for the program due to a busy schedule. With 55,000 individuals experiencing the effects of stroke in British Columbia, Canada[17] and approximately 15 % of them have moderate to mild UE impairment,[18] we estimated that potential participants were 8,250. The program reached approximately 0.15 % (N=12) of the potential target population. Twelve individuals registered for the virtual GRASP program, and 11 out of the 12 consented to participate in the research study. Two participants were excluded from the analysis as they withdrew from the program after week three due to fatigue resulting from other medical conditions (N=1) and after week two due to a change in availability of the caregiver to assist in computer access to the virtual class (N=1). Participant demographic and baseline characteristics for the remaining 9 participants (65.90 ±14.40 years old, 4 females/5 males) are shown in Table 3.

**[H2]** *Effectiveness – Effects of the GRASP program on participants' outcomes*

Table 4 presents the statistical results of clinical outcomes across time points. Nine participants completing the 10-week program were included in the analysis. There were significant differences across timepoints in Arm Capacity and Movement Test (ArmCAM) ($\chi^2(2) = 7.032$, $p = .030$), Rating of Everyday Arm-use in the Community and Home Scale (REACH Scale) ($\chi^2(2) = 6.125$, $p = 0.047$), Stroke Impact Scale (SIS)-Hand ($\chi^2(2) = 10.80$, $p = .005$), and SIS-Hand Recovery ($\chi^2(2) = 12.071$, $p = .002$). Post hoc analysis revealed that there were significant improvements between the baseline and post-test in ArmCAM ($Z = 2.414$, $p = 0.016$), SIS-Hand ($Z = -2.184$, $p = .029$), and SIS-Hand Recovery ($Z = -2.527$, $p = .012$). Significant improvements were also found between the baseline and follow-up in REACH Scale ($Z = -2.00$, $p = 0.046$), SIS-Hand ($Z = -2.694$, $p = .007$), and SIS-Hand Recovery ($Z = -2.375$, $p = .018$).

Individual changes in ArmCAM, SIS-Hand, and SIS-Hand Recovery are shown in the Figure. The MCID of the SIS-hand has been found to be as low as 5.8 (distribution-based estimate) or as high as 17.8 (anchor-based estimate), with 6 of the 9 participants reaching the MCID with the distribution-based estimate and 2 for the anchor-based estimate [19]. A 10% to 15% change in the recovery scale has also been noted as clinically important change[19] and 6 of the 9 participants reach this change. Four of the 9 participants showed meaningful changes in affected UE use measured by the REACH Scale (increased one level). Two participants showed a decrease over time with the ArmCam, but both showed improvements with the SIS-Hand at follow-up, demonstrating the potential discrepancies between measured function and patient-report values.

*Adoption –factors affecting program uptake (staff level) and ongoing participation (individual level)*

**[H3]** Staff level: As shown in Table 5, all staff (N=4) agreed that the implementation was well-planned, the screening procedure was able to select appropriate participants, and the feedback from the audit results was helpful. All staff (N=4), the instructor, volunteers, and coordinator felt comfortable using the videoconferencing technology to deliver the program despite some technical difficulties (eg, inability to see and hear participants easily). While using the videoconferencing technology, all staff (N=4) agreed that the participants could easily see and hear the staff, three staff agreed that they could hear the participants easily, and one staff agreed that she could easily see the participants. The virtual format of the GRASP program generally worked very well reported by the instructor, volunteers, and coordinator. Some advantages of delivering the program via videoconferencing included the "share screen" feature allowing to show videos and pictures easily for demonstrating proper exercise forms and the "breakout rooms" feature to separate participants into small groups for exercise modification and progression. The online format also allowed the program to be accessible to people from a wide geographical range. One common issue raised by the instructor/volunteers was that the instructor/volunteers had difficulties observing some participants' UE movements via videoconferencing as some participants did not have ideal setups (eg, participants using a tablet/phone struggled with adjusting the webcam angle). Another challenge noted by one volunteer was that multiple people talking simultaneously could be distracting and challenging for other participants to join the discussion, especially for people with aphasia. The instructor commented that it was challenging to explain how to fill out the progress tracker in the manual via videoconferencing. As the virtual format introduced technology challenges, one volunteer noted that it therefore slowed down the class and took time away from the exercise and discussions. Besides the challenges from technology, one volunteer noted that progressing exercises to increase the difficulty for one participant with very high UE function was challenging. The coordinator found that the recruitment took longer than expected to fill all spots.

**[H3]** *Individual level:* Common reasons that facilitated ongoing participation included that the participants liked the group (N=7), they perceived improvements (N=7), and the instructor was encouraging (N=7) and well-trained (N=6). When asked what the best part of the class was, six participants identified positive group dynamics, for example: "Learning from each instructor and each other participants. Amazing how much I learned and get new ideas from the others." and "I think the best part of the class was the constant insistence that we use the affected arm, that we found new ways of using it, and we were encouraged to share ideas and bring one or two new ideas to class every week." Another participant reported that "Our instructor was a pleasure to deal with, and she was always encouraging us even in our smallest accomplishments." Three participants identified fatigue, two participants reported pain, and two participants thought the exercises were too difficult, as the barriers to doing the GRASP exercises. All participants agreed that they had the necessary equipment to do the GRASP exercises. Only one participant stated the cost of the program was a barrier and financial assistance was provided by the nonprofit organization. All but one participant was comfortable using the videoconference technology (eg, Zoom software, webcam, computer/tablet/phone, etc.) and identified that the videoconference technology did not interfere with the GRASP program.

**[H2]** *Implementation – Adaptions made during the implementation, fidelity (program level), and participants' adherence (individual level)*

**[H3]** <u>Program level.</u> The virtual GRASP program was implemented successfully by the organization with support from the research team. The program was implemented as intended as verified by the fidelity checklist. The program consisted of daily GRASP exercise self-managed at home, plus weekly discussion of how participants used their affected UE in the previous week, review of weekly log sheets, discussion on facilitator/barriers to doing exercise, break-out sessions to view exercises and progressions, and set goals for the affected UE use in the upcoming week. Some accommodations were made for participants with aphasia, such as asking clear questions, calling on them to give them the floor to speak, being patient, and not interrupting/finishing their sentences. Adaptions made for each RE-AIM category are presented in Table 2. In addition, the following practices were implemented to ensure the confidentiality of weekly videoconferencing meetings: 1) The meetings were hosted on the organization's Zoom account for security, 2) The meeting link was only provided to the participants and their caregivers; 3) The participants and their caregivers were told not to share the meeting link with anyone else; 4) Every meeting is password-protected; 5) Waiting Room feature was used to prevent uninvited guests from joining.

**[H3]** <u>Individual level.</u> Only two participants missed one class out of ten classes, and all other participants (N = 7) had full attendance. The average GRASP practice time per day ranged from 58 mins to 75.7 mins (mean = 63.3, SD = 5.37). Two caregivers were available to attend the weekly classes and assist the participants in various ways, such as managing technology, setting up the GRASP equipment, being supportive and encouraging, and making sure that the exercises were challenging and done correctly.

**[H2]** *Maintenance – The extent to which the program becomes part of routine practices & adaptions made for future programs*

The SRABC/MODC continued to offer the program. Another 10-week iteration (Oct-Dec 2020) has been completed and the screening for the next program (Jan-Mar 2021) has been undergoing. A video describing the virtual program was made by the research team to facilitate the promotion and advertisement (https://youtu.be/lnXKSNXpc8U). Visual cue cards (eg, the microphone card to mute and unmute, the camera card to start or stop their own video, etc.) were developed by the SRABC/MODC regional coordinator for future programs to help participants get familiar with videoconferencing. The program fee was subject to change for future programs to cover an increase in time to screen participants via videoconferencing.

**[H1] Discussion**

The study demonstrated successful implementation of a virtual UE exercise program (i.e., virtual GRASP) delivered via videoconferencing in a group setting for individuals with stroke. By using the RE-AIM framework to evaluate the impact of this implementation, we demonstrated the effectiveness of the program on improving UE function and self-perceived hand function and recovery. Next steps to inform the future delivery of this virtual program were also identified. These findings are valuable to facilitate the broader implementation of this virtual program and guide the field of community-based telerehabilitation, especially during the COVID-19 pandemic, where measures such as social distancing were imposed to limit person-to-person contacts.

The findings of this study demonstrated that the virtual GRASP program had similar effects on improving self-perceived hand function as the in-person GRASP program in our previous study. As the evaluation was conducted virtually via videoconferencing, commonly used clinical assessments (eg, Fugl-Meyer Assessment, Action Research Arm Test, grip strength)

requiring special equipment (eg, a dynamometer, various sizes of blocks) or the examiner to palpate or give resistance were not used in this study. Thus, a newly developed assessment, the ArmCAM test,[15] designed to evaluate UE function remotely through videoconferencing was used. While not all comparisons were significant, medium to large effect sizes were found. Given the sample size is small, it is possible that the study is underpowered, which reduces the likelihood of detecting a true effect.

Similar to our previous study[11] implementing the in-person GRASP community program at a local community center, the following essential components were also found for successful implementation of the virtual format of the GRASP program: the program structure, accessible resources, positive group dynamics, and high program fidelity. Moreover, the virtual format was more accessible geographically compared to the in-person program with the ability to *Reach* to individuals who would not be able to come to weekly in-person classes such as people living in remote areas and in different provinces. The attendance was higher than the in-person GRASP community program, possibly due to less commute stress. Other advantages related to videoconferencing technology may also contribute to the successful implementation, including the use of breakout rooms to allow for individualized exercises progression in smaller groups and the screen sharing to allow for sharing exercises videos/pictures efficiently.

While the videoconferencing technology enabled the GRASP program to be delivered in a virtual format, it also introduced challenges associated with technical difficulties and therefore disrupted the class content agenda. As the SRABC/MODC staff had previous experience in delivering the in-person GRASP community program, factors affecting program uptake reported by staff were primarily due to its adaptions to the virtual format. The most-reported challenge by the instructor and volunteers was the nonideal and limited webcam views for them to observe participants' UE movements, especially when using a monitor with a built-in webcam or using a phone/tablet which requires a holder to place the device at the ideal angle. Although an egg carton holder (Suppl. Appendix 4) was able to place the phone properly, it required more time to adjust the webcam for a clear view. Therefore, future delivery of the program should familiarize the participants with their own videoconferencing setup, which should be done prior to the first class or in the first class. Another challenge was to manage the class when multiple participants were talking simultaneously as it could be distracting, especially for people with aphasia to understand and join the conversation. Videoconferencing etiquette should be introduced in the first class and brought up again when needed. For example, be respectful and do not interrupt other people when they are speaking, physically raise your hand if you want to speak, avoid side conservations, and allow the instructor to mute participants if needed manage to background noise, etc.

**[H2]** *Limitations*

The study has several limitations. First, this is a study from two iterations in one setting. Additional insights may arise with more iterations or with scaling up of the programs. Second, correction for multiple comparisons was not applied as it may cause to miss out on potential differences with the small sample size and low power. Future studies with a larger sample size and multiple comparison correction are needed to confirm our findings. Furthermore, our study excluded individuals who were not able to log into a videoconference to attend the class, which also limited the generalizability. However, we demonstrated a high level of *Reach* to individuals with stroke in remote areas and other provinces, showing the potential of scaling up this virtual GRASP program.

## [H1] Conclusions

This study demonstrated the feasibility of implementing the virtual format of the GRASP program delivered via videoconferencing by a nonprofit organization. We also demonstrated the effectiveness of this virtual program on improving UE motor function and self-perceived hand function in a small sample of individuals with stroke. By evaluating each RE-AIM component, the study provided useful insights to inform the next steps for future iterations and scaling-up and, most importantly, to guide the field of telerehabilitation during the COVID-19 pandemic and beyond.

## Author Contributions

Concept/idea/research design: C-L. Yang, J.J. Eng

Writing: C-L. Yang

Data collection: C-L. Yang

Data analysis: C-L. Yang

Project management: C-L. Yang

Fund procurement: J.J. Eng

Consultation (including review of manuscript before submitting): C-L. Yang, S. Waterson, J.J. Eng,

## Acknowledgments

We gratefully acknowledge the research participants and staff from SRABC/MODC for their support of this project.

## Ethics Approval

All study procedures were approved by the University of British Columbia Behavioural Research Ethics Board, study number H18-03101.

## Funding

This research was supported by the Heart and Stroke Foundation Canadian Partnership for Stroke Recovery (Post-doctoral award to CY), Canadian Institutes of Health Research (Operating Grant FDN 143340 to JJE) and Canada Research Chair Program (JJE).

Table 1. The TIDieR (Template for Intervention Description and Replication) Checklist for Reporting of the Virtual GRASP Program

| | |
|---|---|
| **BRIEF NAME** | Virtual Graded Repetitive Arm Supplementary Program (GRASP) for individuals with stroke |
| **WHY** | Self-administered UE rehabilitation intervention based on intensive, repetitive, and task-specific practice |
| **WHAT** | Materials: Resources such as the instructor manual, participant manual, and videos can be accessed at https://neurorehab.med.ubc.ca/grasp/.<br>Procedures:<br>• The program consisted of weekly group classes delivered via videoconferencing and individualized homework exercises.<br>• The typical schedule of a weekly class started with 15 mins of experience sharing and discussion, followed by 35 mins of exercise progression and modification, and ended with 10 mins of goal setting and wrap-up (see details in Suppl. Appendix 3). |
| **WHO PROVIDED** | • The SRABC/MODC regional coordinator provided operational and logistical oversight including in-kind promotion, administration, instructors, and volunteers recruitment, supervision of the instructor, and set cost-recovery fees to cover the salary of the instructor.<br>• The virtual GRASP program instructor screened potential participants, led the virtual program, and supervised and trained the volunteers. The instructor had experience leading the GRASP in-person community program.<br>• Two to three volunteers were available to assist with the classes under the supervision by the program instructor. One volunteer was a registered kinesiologist, and the other two volunteers were health science students. |
| **HOW and WHERE** | The program was delivered online via video conferencing |
| **WHEN and HOW MUCH** | 10 one-hour weekly classes in a group setting plus one hour of individualized daily exercises. The program fee was $85 CAD. |
| **TAILORING** | The exercises were modified and progressed for each participant in the weekly classes. |
| **MODIFICATIONS** | N/A |
| **HOW WELL** | Two audits were conducted by the research team member during in-class observation via videoconferencing in the 2$^{nd}$ and 6$^{th}$ weeks and the audit feedback was then provided to the instructor. |

Table 2. RE-AIM Measures and Data Sources and Adaptations for Virtual Delivery[a]

| Assessment Level | Measures | Data Sources | Timeline | Adaptations |
|---|---|---|---|---|
| Reach | • Recruitment rate<br>• Participant demographic | • Screening and enrollment log sheet (Instructors)<br>• Intake form (Participants with stroke) | Pre-program<br>Baseline | Screening done via Zoom and payment done electronically as previously both were done in person at a local community center. Group size was reduced from 8 to 6 participants to minimize interference during group discussion via videoconferencing. |
| Effectiveness [b] | • ArmCAM<br>• REACH<br>• SIS-Hand<br>• SIS-Hand Recovery | • Clinical assessments (Participants with stroke) | Baseline, post-test, follow-up | All outcomes were done via videoconferencing, rather than in-person. |
| Adoption | • Satisfaction and challenges of the program | • Staff survey (Instructors and coordinator)<br>• Exit survey (Participants with stroke) | Immediately post-program | Participants with stroke filled out an online exit survey, rather than filling out a paper survey at a local community center. |
| Implementation | | | | Videos and pictures on correct ways to do exercises were shared via videoconferencing. Break-out rooms with a maximum of 2 participants in one break-out room were used to view exercises and progressions in smaller groups. Participants showed their weekly log sheets to the group via videoconferencing |
| *Program level* | • Fidelity checklist | • Audit record (University researchers) | In-class ($2^{nd}$ and $6^{th}$) observations | |
| | • Adaptions made | • The University researchers and SRABC/MODC staff | During-study | |
| | • Program attendance rate | • Attendance record (Instructor) | During-program | |
| *Individual level* | • GRASP practice time | • GRASP log sheet (Participants with stroke) | | |

| | | | | |
|---|---|---|---|---|
| | | | | as accountability. |
| Maintenance | • Extent to which the GRASP program is intended to be sustained over time<br>• Adaptions made for future programs | • Next implementation planning (The University researchers and SRABC/MODC staff) | Immediately post-program | |

[a]ArmCAM = Arm Capacity and Movement test [15]; REACH = The Rating of Everyday Arm-use in the Community and Home Scale [20]; SIS= Stroke Impact Scale [21].

[b]The effectiveness data collected from the participants with stroke was done by one university researcher who was not involved in delivering the GRASP program to the participants.

Table 3. Stroke Participant Characteristic (N=9)[a]

| Characteristic | |
|---|---|
| Age, y, mean ± SD [Range] | 65.90 ±14.40 [39.87 – 83.30] |
| Sex, F/M | 4 F / 5 M |
| Ethnicity, Asian/Caucasian/European | 3/4/2 |
| Province, British Columbia/Alberta/Nova Scotia | 7/1/1 |
| Time Poststroke, months, mean ± SD [Range] | 65.86 ± 111.15 [7.90 – 348.40] |
| Side of Paresis, L/R | 4 L / 5 R |
| Dominant Side, L/R | 1 L / 8 R |
| Device used to log into weekly videoconferencing classes Desktop/Laptop/Tablet/Phone | 3/4/1/1 |
| Participants with aphasia | 2 |
| Previous experience with in-person GRASP program | 2 |
| Caregiver available to support during the group classes | 2 |

[a] The data were collected from 2 GRASP programs delivered over the same period of 10 weeks. There were five participants and four participants in these two programs, respectively.

Table 4. Mean ± SD and Statistical Results of Clinical Outcomes (N=9)

| Clinical Outcomes | Baseline | Post-test | Follow-up | Effect size | |
|---|---|---|---|---|---|
| | | | | Baseline vs. Post-test | Baseline vs. Follow-up |
| **ArmCAM (/30)** [bc] | 18.67 ± 8.23 | 20.00 ± 8.54 | 19.78 ± 8.32 | 0.57 | 0.40 |
| **REACH (0-5)** [bd] | 3.00 ± 0.71 | 3.56 ± 0.73 | 3.44 ± 0.88 | 0.45 | 0.47 |
| **SIS-Hand (/100)** [bcd] | 35.56 ± 21.40 | 47.56 ± 21.30 | 50.22 ± 22.73 | 0.51 | 0.63 |
| **SIS-Hand Recovery (/100)** [bcd] | 46.67 ± 19.84 | 60.56 ± 20.83 | 58.78 ± 20.83 | 0.60 | 0.60 |

[a]ArmCAM = Arm Capacity and Movement Test; REACH = The Rating of Everyday Arm-use in the Community and Home Scale; SIS = Stroke Impact Scale.

[b]Indicate significant differences across time points.

[c]Indicate significant improvement between baseline and post-test.

[d]Indicate significant improvement between baseline and follow-up.

Table 5. The Staff's (N=4) and Participants' (N=9) Responses (%) to the Statements Related to Implementation and Videoconferencing Technology in the Staff and Participant Surveys

| Statements | Strongly agree/ Agree | Neutral | Disagree/ Strongly Disagree |
|---|---|---|---|
| **Staff** | | | |
| **Implementation** | | | |
| I think the implementation of the GRASP program was well-planned. | 4 | 0 | 0 |
| I think the screening procedure was able to select appropriate people with stroke for the GRASP program. | 4 | 0 | 0 |
| I think the feedback from the audit is helpful. | 4 | 0 | 0 |
| **Videoconferencing technology** | | | |
| I felt comfortable using the videoconference technology to deliver the GRASP program. | 4 | 0 | 0 |
| The participants could easily see me while using the videoconference technology. | 4 | 0 | 0 |
| The participants could easily hear me while using the videoconference technology. | 4 | 0 | 0 |
| I could easily see the participants while using the videoconference technology | 1 | 2 | 1 |
| I could easily hear the participants while using the videoconference technology. | 3 | 1 | 0 |
| The videoconference technology did not interfere with the GRASP program. | 2 | 1 | 1 |
| **Participant** | | | |
| **Videoconferencing technology** | | | |
| I felt comfortable using the videoconference technology. | 8 | 0 | 1 |
| The instructor could easily see me while using the videoconference technology. | 9 | 0 | 0 |
| The instructor could easily hear me while using the videoconference technology. | 9 | 0 | 0 |
| I could easily see the instructor while using the videoconference technology. | 9 | 0 | 0 |
| I could easily hear the instructor while using the videoconference technology. | 8 | 0 | 1 |

**Figure legend**

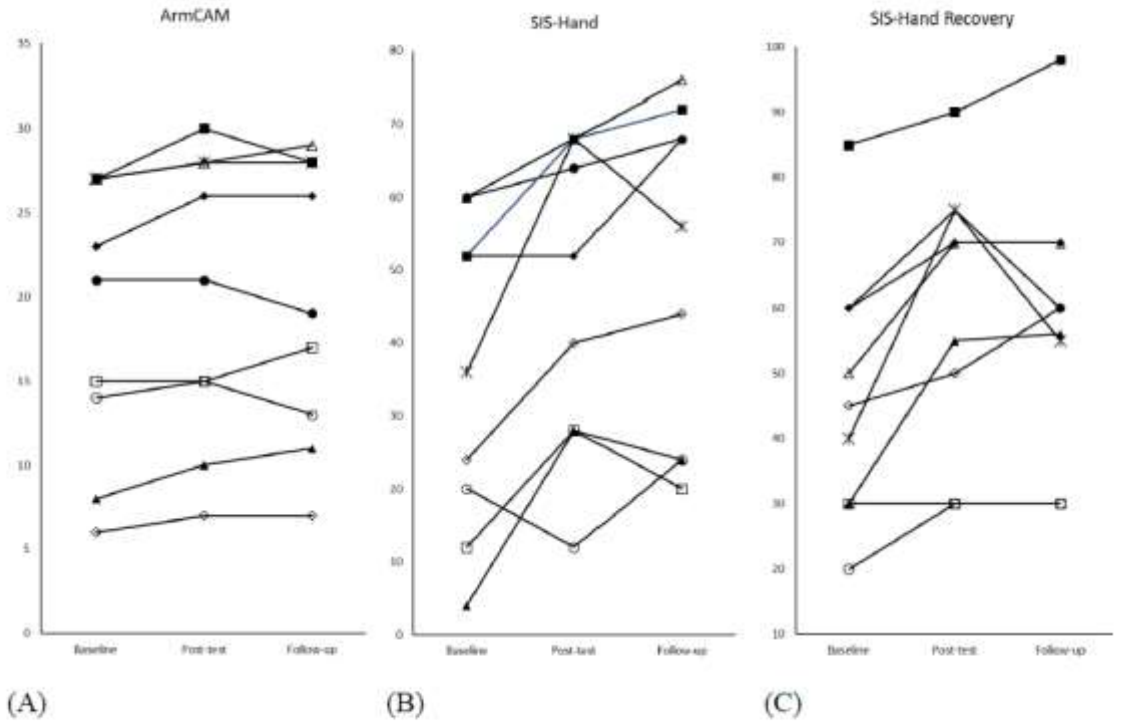

Figure. Individual scores for baseline, post-test, 2-month follow-up are shown for (A) ArmCAM, (B) SIS-Hand, (C) SIS-Hand Recovery. ArmCAM, Arm Capacity and Movement test; SIS, Stroke Impact Scale.

UNCORRECTED
