# Implementation and Evaluation of the Virtual Graded Repetitive Arm Supplementary Program (GRASP) for Individuals With Stroke During the COVID-19 Pandemic and Beyond

**DOI:** 10.1093/ptj/pzab083

**Published:** 2021-03-04

**Authors:** Chieh-ling Yang, Seonaid Waterson, Janice J Eng

**Affiliations:** Department of Physical Therapy, University of British Columbia, Vancouver, British Columbia, Canada; Rehabilitation Research Program, Vancouver Coastal Health Research Institute, Vancouver, British Columbia, Canada; Stroke Recovery Association of British Columbia, March of Dimes Canada, Vancouver, British Columbia, Canada; Department of Physical Therapy, University of British Columbia, Vancouver, British Columbia, Canada; Rehabilitation Research Program, Vancouver Coastal Health Research Institute, Vancouver, British Columbia, Canada

**Keywords:** Arm, Hand, Exercise, Implementation, RE-AIM, Stroke, Telerehabilitation

## Abstract

**Objective:**

Given the uncertainty of the coronavirus disease 2019 (COVID-19) pandemic, implementing telerehabilitation that enables the remote delivery of rehabilitation services is needed to mitigate the spread of COVID-19. We studied the implementation and the effectiveness of the virtual Graded Repetitive Arm Supplementary Program (GRASP) delivered and evaluated via videoconferencing in individuals with stroke.

**Methods:**

The Reach, Effectiveness, Adoption, Implementation, Maintenance (RE-AIM) framework with mixed methods was used to evaluate the implementation of the 2 iterations of the program delivered by a nonprofit organization during the pandemic.

**Results:**

*Reach*: Seventeen people were screened, 13 people were eligible, and 11 consented to participate in the study. *Effectiveness*: Between baseline and posttest, participants with stroke demonstrated significant improvement in upper extremity function (Arm Capacity and Movement Test) and self-perceived upper extremity (UE) function (Stroke Impact Scale). *Adoption*: Factors that facilitate program uptake by the staff were well-planned implementation, appropriate screening procedure, and helpful feedback from the audits. All staff felt comfortable using videoconferencing technology to deliver the program despite some technical difficulties. Factors contributing to ongoing participation included that the participants liked the group, they perceived improvements, and the instructor was encouraging. Only one participant with stroke was not comfortable using the videoconferencing technology. *Implementation*: The program was implemented as intended as evaluated by a fidelity checklist. Participants’ adherence was high, as verified by the average attendance and practice time. *Maintenance*: The organization continued to offer the program.

**Conclusion:**

The virtual GRASP program was successfully implemented. Although the program was effective in improving both measured and perceived UE function in a small sample of individuals with stroke, caution should be taken in generalizing the results.

**Impact:**

Implementing telerehabilitation is crucial to optimize patient outcomes and reduce the spread of COVID-19. Our findings provide guidance on the process of delivering a UE rehabilitation program remotely via videoconferencing for stroke. Moreover, insights that arise from this study also inform the implementation of other telerehabilitation services.

## Introduction

The World Health Organization announced coronavirus disease 2019 (COVID-19) as a pandemic in March 2020.^1^ Based on some mathematical models, the overall duration of the COVID-19 pandemic could last into 2022, with the possibility of a resurgence in contagion as late as 2024.^2^ As COVID-19 is transmitted from person to person, rehabilitation services that can be delivered remotely via communication technologies (ie, telerehabilitation)^3^ without in-person contact are needed to mitigate the spread of COVID-19. While telerehabilitation has some key advantages such as eliminating the need for in-person contact and travel, and increased accessibility for people in rural and remote areas, the challenges associated with telerehabilitation have been well documented.^4^ Some common challenges included the inability to conduct hands-on treatment and the lack of expertise to troubleshoot the communication technology.^5^^,^^6^ Thus, in times of COVID-19 and beyond, understanding how to implement and deliver a virtual rehabilitation exercise program is a matter of priority.

As approximately 88% of individuals with stroke did not regain complete functional recovery in their upper extremity (UE) at 6 months poststroke,^7^ stroke survivors often need continuous rehabilitation services to improve their UE function after being discharged home. The Graded Repetitive Arm Supplementary Program (GRASP) is a self-administered UE rehabilitation intervention designed for individuals with stroke based on intensive, repetitive, and task-specific practice.^8^^,^^9^ It has been suggested by the Canadian Stroke Best Practice Recommendations for UE management.^10^ Our recent study showed the effectiveness and successful implementation of the GRASP as a community program in a group setting (ie, GRASP community program) delivered by a local nonprofit organization (Stroke Recovery Association of British Columbia, an affiliate of the March of Dimes Canada; SRABC/MODC) at a local community center.^11^ The GRASP community program consisted of 10 weekly 1-hour group classes and individualized homework exercises. The organization had delivered the in-person GRASP community program in a local community center since April 2019. However, owing to the COVID-19 pandemic and related policies for social distancing, the organization decided to adapt the in-person GRASP community program into a virtual program (ie, virtual GRASP program) delivered via videoconferencing.

The purpose of this study was to evaluate the implementation process from the 2 iterations of the virtual GRASP program delivered via videoconferencing by a nonprofit organization using the Reach, Effectiveness, Adoption, Implementation, and Maintenance (RE-AIM) framework. The findings of this study will not only inform the considerations of the successful implementation of the virtual GRASP program but also allow for insight into the delivery of other telerehabilitation programs during the COVID-19 pandemic and beyond.

## Methods

### Implementation Overview

Two virtual GRASP programs were delivered from June to August 2020. The 2 programs were delivered over the same period of 10 weeks but on different days during the week. Promotion and participant screening began 1 month prior to the commencement of the program. To ensure the program fidelity, 2 audits were conducted by the research team member during in-class observation via videoconferencing during the second and sixth weeks and the audit feedback was then provided to the instructor. The research team, as an outreach facilitator,^12^ provided support and consultation to assist transforming the in-person community program to a virtual format delivered by the SRABC/MODC. The SRABC/MODC regional coordinator provided operational and logistical oversight including in-kind promotion, administration, instructors, recruitment of volunteers, supervision of the instructor, and set cost-recovery fees to cover the salary of the instructor. The virtual GRASP program instructor screened potential participants, led the virtual program, and supervised and trained the volunteers. The virtual GRASP program volunteers assisted with classes under the supervision by the program instructor. The roles of the research team and SRABC/MODC are summarized in Supplementary Appendix 1.

### Virtual Graded Repetitive Arm Supplementary Program

The virtual GRASP program was adapted from the GRASP community program (http://neurorehab.med.ubc.ca/grasp/). Details regarding the virtual program are summarized using the Template for Intervention Description and Replication checklist^3^ in Table 1. The program consisted of 10 weekly 1-hour group classes delivered via videoconferencing using Zoom software (Zoom Video Communications Inc, 2016) and individualized homework exercises. Participants were responsible for completing 1 hour of GRASP exercises daily and logging the practice time in the manual (Suppl. Appendix 2). If available, participants were encouraged to have their caregivers of family members attend the weekly class. The program fee was CAD $85, reflecting the cost of staffing and manuals. The typical schedule of a weekly class started with 15 minutes of experience sharing and discussion, followed by 30 minutes of exercise progression and modification, and ended with 15 minutes of goal setting and wrap-up (Suppl. Appendix 3). General principles of exercise progression and modification were outlined in the GRASP instructor manual (https://neurorehab.med.ubc.ca/grasp/grasp-manuals-and-resources/). One instructor led the weekly class, and 2 or 3 volunteers were available to assist with the classes. Before the program started, the participant manuals were mailed to the participants. The participants were responsible to source their own equipment for the program according to an equipment list provided by the SRABC/MODC. Most of the equipment was available in the home except for weights, therapy putty, and hand grippers. Information on where to purchase the equipment and alternatives (eg, play dough to substitute therapy putty) was provided to the participants. To ensure the safety of participants during online classes, the participants were asked to leave their cameras on so that the staff could visually monitor participants’ conditions during online classes. The emergency contact information, home address, and phone number of each participant were on hand in case of an emergency or medical event. If the staff were concerned about the participant’s safety, the staff would contact the person by phone and offer assistance by calling 911 or his or her emergency contact person. The instructor also reviewed the program safety guideline (ie, follow the instructions provided by the instructor) with the participants during the screening process.

**Table 1:** Template for Intervention Description and Replication Checklist for Reporting of the Virtual Graded Repetitive Arm Supplementary Program^a^

**Item**	**Details**
Brief name	Virtual GRASP for individuals with stroke
Why	Self-administered UE rehabilitation intervention based on intensive, repetitive, and task-specific practice
What	Materials: Resources such as the instructor manual, participant manual, and videos can be accessed at https://neurorehab.med.ubc.ca/grasp/. Procedures: • The program consisted of weekly group classes delivered via videoconferencing and individualized homework exercises. • The typical schedule of a weekly class started with 15 min of experience sharing and discussion, followed by 35 min of exercise progression and modification, and ended with 10 min of goal setting and wrap-up (see details in Suppl. Appendix 3).
Who provided	• The SRABC/MODC regional coordinator provided operational and logistical oversight including in-kind promotion, administration, instructors, and volunteers recruitment, supervision of the instructor, and set cost-recovery fees to cover the salary of the instructor. • The virtual GRASP program instructor screened potential participants, led the virtual program, and supervised and trained the volunteers. The instructor had experience leading the GRASP in-person community program. • Two or 3 volunteers were available to assist with the classes under the supervision by the program instructor. One volunteer was a registered kinesiologist, and the other 2 volunteers were health science students.
How and where	The program was delivered online via video conferencing
When and how much	Ten 1-h weekly classes in a group setting plus 1 h of individualized daily exercises. The program fee was CAD $85.
Tailoring	The exercises were modified and progressed for each participant in the weekly classes.
Modifications	NA
How well	Two audits were conducted by the research team member during in-class observation via videoconferencing in the second and sixth weeks and the audit feedback was then provided to the instructor.

^*a*^CAD = Canadian dollars; GRASP = Graded Repetitive Arm Supplementary Program; NA = not available; SRABC/MODC = Stroke Recovery Association of British Columbia, affiliate of the March of Dimes Canada; UE = upper extremity.

### Participants With Stroke

Individuals with stroke interested in attending the program contacted the SRABC/MODC coordinator and then were screened via videoconferencing by the program instructor. Eligibility criteria were as follows: (1) age ≥ 19 years; (2) diagnosis of stroke; (3) difficulty using the affected UE; (4) some voluntary movement in the affected UE, including the ability to lift the affected arm to the chest level and hold for 5 seconds, some ability to extend the affected wrist, and some ability to grasp and release an object such as a cup handle; 5) living in the community; 6) ability to log into a videoconference to attend the weekly class; 7) ability to understand and follow instructions; and 8) willingness to practice GRASP exercises daily. Eligibility criterion 4 was assessed by asking potential participants to lift the affected arm to the chest level (approximately 60–70 degrees of shoulder flexion), extend the affected wrist (visible active wrist extension from a flexion position), and to grasp and release an object (eg, a cup handle, a block, a pill bottle). Participants with severe pain that prevented movement in the affected arm and hand were excluded. Eligible participants could register for the program. At that point, they were approached to participate and consent to an optional research study by the research team.

### Staff

One paid instructor with experience leading the GRASP community program led the program and had received prior GRASP training through an in-person group workshop led by a physical therapist about a year prior. The instructor was a health science student. One volunteer was a registered kinesiologist, and the other 2 volunteers were health science students. The instructor and volunteers received training on aphasia provided by the organization. The aphasia training consisted of a webinar on aphasia knowledge provided by a speech-language pathologist and an online learning module on Supported Conversation for Adults with Aphasia from the Aphasia Institute (https://www.aphasia.ca/communityhub/).

### Data Collection and Analysis

The implementation was examined using the RE-AIM framework, which provided a systematic and comprehensive framework to evaluate the translation of an evidence-based intervention to practice (http://www.re-aim.org/).^13^ The framework encompasses 5 elements to be assessed (Reach, Effectiveness, Adoption, Implementation, and Maintenance) and it has been widely used to design, implement, and evaluate research.^14^  Table 2 provides an overview of the variables under each RE-AIM element, the data sources used to assess each variable, and the data collection timeline. All data were collected virtually using videoconferencing software (Zoom Video Communications Inc). A newly developed online assessment, Arm Capacity and Movement test (ArmCAM),^15^ was used to assess the body functions and activities according to the International Classification of Functioning, Disability, and Health. The Rating of Everyday Arm-use in the Community and Home Scale (REACH) and the Stroke Impact Scale Hand subscore (SIS-Hand) were used to assess the Activities domain. The ArmCAM has good reliability and validity with the Fugl-Meyer Assessment for upper extremity, Action Research Arm Test, SIS-Hand, and REACH.^15^ The ArmCAM consists of 10 items that measure gross movements (2 items), supported reach (1 item), functional reach and grasp movements (3 items), functional wrist movement (1 item), and fine motor skills (3 items). All items are rated on a 4-point ordinal scale (0, can perform no part of task; 1, perform task partially; 2, complete task, but slower, with difficulty, or using compensatory strategies; 3, perform task normally). Assessment objects include 1 magazine, 1 sheet of paper towel, 2 cups, 1 jar with a lid, 1 soup can, and 3 coins. It takes approximately 20 minutes to administer the test. Scored participant videos of the ArmCAM are available at the https://www.youtube.com/watch?v=CsplVu_U-mg.

**Table 2:** RE-AIM Measures and Data Sources and Adaptations for Virtual Delivery^a^

**Assessment Level**	**Measures**	**Data Sources**	**Timeline**	**Adaptations**
Reach	• Recruitment rate	• Screening and enrollment log sheet (instructors)	Preprogram	Screening held via Zoom and payment accepted electronically as previously both were done in person at a local community center. Group size was reduced from 8 to 6 participants to minimize interference during group discussion via videoconferencing.
	• Participant demographic	• Intake form (participants with stroke)	Baseline	

Effectiveness*^b^*	• ArmCAM • REACH • SIS-Hand • SIS-Hand Recovery	• Clinical assessments (participants with stroke)	Baseline, posttest, follow-up	All outcomes were performed via videoconferencing, rather than in-person.
Adoption	• Satisfaction and challenges of the program	• Staff survey (instructors and coordinator) • Exit survey (participants with stroke)	Immediately postprogram	Participants with stroke filled out an online exit survey, rather than filling out a paper survey at a local community center.
Implementation				Videos and pictures on correct ways to complete exercises were shared via videoconferencing. Breakout rooms with a maximum of 2 participants in one break-out room were used to view exercises and progressions in smaller groups. Participants showed their weekly log sheets to the group via videoconferencing as accountability.
Program level	• Fidelity checklist	• Audit record (university researchers)	In-class (second and sixth) observations	
	• Adaptions made	• University researchers and SRABC/MODC staff	During-study	
	• Program attendance rate	• Attendance record (instructor)	During-program	
Individual level	• GRASP practice time	• GRASP log sheet (participants with stroke)		
Maintenance	• Extent to which the GRASP program is intended to be sustained over time • Adaptions made for future programs	• Next implementation planning (university researchers and SRABC/MODC staff)	Immediately postprogram6 months after the study ended	

^*a*^ArmCAM = Arm Capacity and Movement test^15^; REACH = The Rating of Everyday Arm-use in the Community and Home Scale^20^; SIS = Stroke Impact Scale^21^; SRABC/MODC = Stroke Recovery Association of British Columbia, affiliate of the March of Dimes Canada.

^*b*^Effectiveness data from the participants with stroke were collected by one university researcher who was not involved in delivering the GRASP program to the participants.

Descriptive analysis was used to summarize baseline demographic, stroke characteristics, individual changes in clinical assessment scores, responses to the questionnaires, and program and individual records. Effectiveness shown by the differences across 3 time points (baseline, posttest, follow-up) was assessed by the Friedman tests with a within-group factor of time. Post hoc paired comparisons (baseline vs posttest; baseline vs follow-up) were made using the Wilcoxon signed-rank test. The effect size *r* (small effect: 0.1 < *r* < .3; medium effect: 0.3 < *r* < .5; large effect: *r* ≥ .5) is calculated as a *Z* statistic from the Wilcoxon signed-rank test divided by the square root of the sample size.^16^ The significance level was set as *P* less than .05. SPSS 25.0 software (IBM, Armonk, New York, USA) was used for statistical analyses.

### Role of the Funding Source

The funders played no role in the design, conduct, or reporting of this study.

## Results

### Reach—Size and Representativeness of Enrolled Participants

Seventeen individuals were screened for eligibility and 13 were eligible. Four did not meet the inclusion criteria because of insufficient voluntary movement in the affected arm/hand. Among the 13 eligible individuals, 1 person did not register for the program because of a busy schedule. With 55,000 individuals experiencing the effects of stroke in British Columbia, Canada,^17^ and approximately 15% of them have moderate to mild UE impairment,^18^ we estimated that potential participants were 8250. The program reached approximately 0.15% (N = 12) of the potential target population. Twelve individuals registered for the virtual GRASP program, and 11 out of the 12 consented to participate in the research study. Two participants were excluded from the analysis because they withdrew from the program after week 3 because of fatigue resulting from other medical conditions (N = 1) and after week 2 because of a change in availability of the caregiver to assist in computer access to the virtual class (N = 1). Participant demographic and baseline characteristics for the remaining 9 participants (age 65.90 ± 14.40 years, 4 women/5 men) are shown in Table 3.

**Table 3:** Stroke Participant Characteristic (N = 9)^a^

**Characteristic**	**n**
Age, y, mean (SD), range	65.90 (14.40); 39.87–83.30
Sex, F/M	4 F/5 M
Ethnicity Asian/Caucasian/European	3/4/2
Province British Columbia/Alberta/Nova Scotia	7/1/1
Time poststroke, mo, mean (SD), range	65.86 (111.15); 7.90–348.40
Side of paresis, L/R	4 L/5 R
Dominant side, L/R	1 L/8 R
Device used to log into weekly videoconferencing classes Desktop/laptop/tablet/phone	3/4/1/1
Participants with aphasia	2
Previous experience with in-person GRASP program	2
Caregiver available to support during group classes	2

^*a*^Data were collected from 2 GRASP programs delivered over the same period of 10 weeks. There were 5 participants and 4 participants in these 2 programs, respectively. F = female; GRASP = Graded Repetitive Arm Supplementary Program; L = left; M = male; R = right.

### Effectiveness—Effects of Graded Repetitive Arm Supplementary Program on Participants’ Outcomes

Table 4 presents the statistical results of clinical outcomes across time points. Nine participants completing the 10-week program were included in the analysis. There were significant differences across time points in ArmCAM [χ^2^(2) = 7.032, *P* = .030], REACH Scale [χ^2^(2) = 6.125, *P* = .047], SIS-Hand [χ^2^(2) = 10.80, *P* = .005], and SIS-Hand Recovery [χ^2^(2) = 12.071, *P* = .002]. Post hoc analysis revealed that there were significant improvements between the baseline and posttest in ArmCAM (*Z* = 2.414, *P* = .016), SIS-Hand (*Z* = −2.184, *P* = .029), and SIS-Hand Recovery (*Z* = −2.527, *P* = .012). Significant improvements were also found between the baseline and follow-up in the REACH Scale (*Z* = −2.00, *P* = .046), SIS-Hand (*Z* = −2.694, *P* = .007), and SIS-Hand Recovery (*Z* = −2.375, *P* = .018).

**Table 4:** Mean (SD) and Statistical Results of Clinical Outcomes (N = 9)^a^

**Clinical Outcomes**	**Baseline**	**Posttest**	**Follow-up**	**Effect Size**	
				**Baseline vs Posttest**	**Baseline vs Follow-up**
ArmCAM (/30)*^b^^,^^c^*	18.67 (8.23)	20.00 (8.54)	19.78 (8.32)	0.57	0.40
REACH (0–5)*^b^^,^^d^*	3.00 (0.71)	3.56 (0.73)	3.44 (0.88)	0.45	0.47
SIS-Hand (/100)*^b^^,^^c^^,^^d^*	35.56 (21.40)	47.56 (21.30)	50.22 (22.73)	0.51	0.63
SIS-Hand Recovery (/100)*^b^^,^^c^^,^^d^*	46.67 (19.84)	60.56 (20.83)	58.78 (20.83)	0.60	0.60

^*a*^ArmCAM = Arm Capacity and Movement Test; REACH = The Rating of Everyday Arm-use in the Community and Home Scale; SIS = Stroke Impact Scale.

^*b*^Significant differences across time points.

^*c*^Significant improvement between baseline and posttest.

^*d*^Significant improvement between baseline and follow-up.

Individual changes in ArmCAM, SIS-Hand, and SIS-Hand Recovery are shown in the Figure. The minimal clinically important difference of the SIS-Hand has been found to be as low as 5.8 (distribution-based estimate) or as high as 17.8 (anchor-based estimate), with 6 of the 9 participants reaching the minimal clinically important difference with the distribution-based estimate and 2 for the anchor-based estimate.^19^ A 10% to 15% change in the recovery scale has also been noted as clinically important change^19^ and 6 of the 9 participants reach this change. Four of the 9 participants showed meaningful changes in affected UE use measured by the REACH Scale (increased one level). Two participants showed a decrease over time with the ArmCam, but both showed improvements with the SIS-Hand at follow-up, demonstrating the potential discrepancies between measured function and patient-report values.

**Figure:**
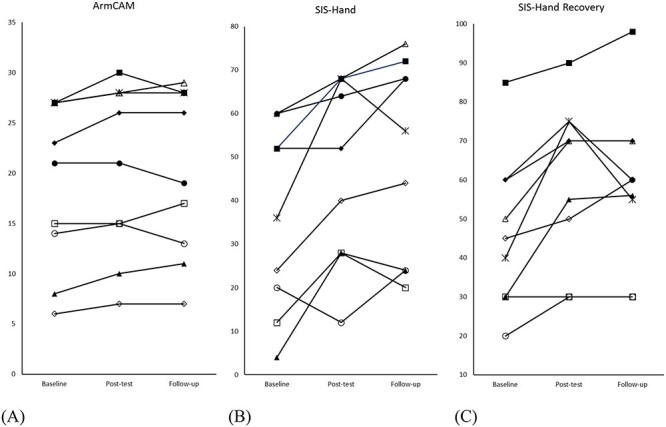
Individual Scores for Baseline, Posttest, 2-Month Follow-up Are Shown for (A) Arm Capacity and Movement test, (B) Stroke Impact Scale (SIS)-Hand, (C) and SIS-Hand Recovery.

### Adoption—Factors Affecting Program Uptake (Staff Level) and Ongoing Participation (Individual Level)

#### Staff Level

As shown in Table 5, all staff (N = 4) agreed that the implementation was well planned, the screening procedure was able to select appropriate participants, and the feedback from the audit results was helpful. All staff (N = 4), the instructor, volunteers, and coordinator felt comfortable using the videoconferencing technology to deliver the program despite some technical difficulties (eg, inability to see and hear participants easily). While using the videoconferencing technology, all staff (N = 4) agreed that the participants could easily see and hear the staff, 3 staff agreed that they could hear the participants easily, and 1 staff agreed that she could easily see the participants. The virtual format of the GRASP program generally worked very well reported by the instructor, volunteers, and coordinator. Some advantages of delivering the program via videoconferencing included the “share screen” feature allowing to show videos and pictures easily for demonstrating proper exercise forms and the “breakout rooms” feature to separate participants into small groups for exercise modification and progression. The online format also allowed the program to be accessible to people from a wide geographical range. One common issue raised by the instructor/volunteers was that the instructor/volunteers had difficulties observing some participants’ UE movements via videoconferencing as some participants did not have ideal setups (eg, participants using a tablet/phone struggled with adjusting the webcam angle). Another challenge noted by one volunteer was that multiple people talking simultaneously could be distracting and challenging for other participants to join the discussion, especially for people with aphasia. The instructor commented that it was challenging to explain how to fill out the progress tracker in the manual via videoconferencing. As the virtual format introduced technology challenges, one volunteer noted that it therefore slowed down the class and took time away from the exercise and discussions. Besides the challenges from technology, one volunteer noted that progressing exercises to increase the difficulty for one participant with very high UE function was challenging. The coordinator found that the recruitment took longer than expected to fill all spots.

**Table 5:** Staff (N = 4) and Participants’ (N = 9) Responses (%) to the Statements Related to Implementation and Videoconferencing Technology in the Staff and Participant Surveys^a^

**Statements**	**Strongly Agree/Agree**	**Neutral**	**Disagree/Strongly Disagree**
Staff			
Implementation			
I think the implementation of the GRASP program was well-planned.	4	0	0
I think the screening procedure was able to select appropriate people with stroke for the GRASP program.	4	0	0
I think the feedback from the audit is helpful.	4	0	0
Videoconferencing technology			
I felt comfortable using the videoconference technology to deliver the GRASP program.	4	0	0
The participants could easily see me while using the videoconference technology.	4	0	0
The participants could easily hear me while using the videoconference technology.	4	0	0
I could easily see the participants while using the videoconference technology	1	2	1
I could easily hear the participants while using the videoconference technology.	3	1	0
The videoconference technology did not interfere with the GRASP program.	2	1	1
Participant			
Videoconferencing technology			
I felt comfortable using the videoconference technology.	8	0	1
The instructor could easily see me while using the videoconference technology.	9	0	0
The instructor could easily hear me while using the videoconference technology.	9	0	0
I could easily see the instructor while using the videoconference technology.	9	0	0
I could easily hear the instructor while using the videoconference technology.	8	0	1

^*a*^GRASP = Graded Repetitive Arm Supplementary Program.

#### Individual Level

Common reasons that facilitated ongoing participation included that the participants liked the group (N = 7), they perceived improvements (N = 7), and the instructor was encouraging (N = 7) and well trained (N = 6). When asked what the best part of the class was, 6 participants identified positive group dynamics, for example: “Learning from each instructor and each other participants. Amazing how much I learned and get new ideas from the others” and “I think the best part of the class was the constant insistence that we use the affected arm, that we found new ways of using it, and we were encouraged to share ideas and bring one or two new ideas to class every week.” Another participant reported that “Our instructor was a pleasure to deal with, and she was always encouraging us even in our smallest accomplishments.” Three participants identified fatigue, 2 participants reported pain, and 2 participants thought the exercises were too difficult, as the barriers to doing the GRASP exercises. All participants agreed that they had the necessary equipment to do the GRASP exercises. Only one participant stated the cost of the program was a barrier and financial assistance was provided by the nonprofit organization. All but one participant was comfortable using the videoconference technology (eg, Zoom software, webcam, computer/tablet/phone) and identified that the videoconference technology did not interfere with the GRASP program.

### Implementation—Adaptions Made During Implementation, Fidelity (Program Level), and Participants’ Adherence (Individual Level)

#### Program Level

The virtual GRASP program was implemented successfully by the organization with support from the research team. The program was implemented as intended as verified by the fidelity checklist. The program consisted of daily GRASP exercise self-managed at home, plus weekly discussion of how participants used their affected UE in the previous week, review of weekly log sheets, discussion on facilitator/barriers to doing exercise, breakout sessions to view exercises and progressions, and set goals for the affected UE use in the upcoming week. Some accommodations were made for participants with aphasia, such as asking clear questions, calling on them to give them the floor to speak, being patient, and not interrupting/finishing their sentences. Adaptions made for each RE-AIM category are presented in Table 2. In addition, the following practices were implemented to ensure the confidentiality of weekly videoconferencing meetings: 1) the meetings were hosted on the organization’s Zoom account for security, 2) the meeting link was provided only to the participants and their caregivers; 3) the participants and their caregivers were told not to share the meeting link with anyone else; 4) every meeting is password protected; and 5) the waiting room feature was used to prevent uninvited guests from joining.

#### Individual Level

Only 2 participants missed 1 class out of 10 classes, and all other participants (N = 7) had full attendance. The average GRASP practice time per day ranged from 58 minutes to 75.7 minutes (mean = 63.3, SD = 5.37). Two caregivers were available to attend the weekly classes and assist the participants in various ways, such as managing technology, setting up the GRASP equipment, being supportive and encouraging, and making sure that the exercises were challenging and performed correctly.

### Maintenance—Extent to Which Program Becomes Part of Routine Practices and Adaptions Made for Future Programs

The SRABC/MODC continued to offer the program. Another 2 10-week iterations (October to December 2020 and January to March 2021) have been completed. The next program (March to June 2021) has been undergoing. A video describing the virtual program was made by the research team to facilitate the promotion and advertisement (https://youtu.be/lnXKSNXpc8U). Visual cue cards (eg, microphone card to mute and unmute, camera card to start or stop their own video) were developed by the SRABC/MODC regional coordinator for future programs to help participants get familiar with videoconferencing. The program fee was subject to change for future programs to cover an increase in time to screen participants via videoconferencing.

## Discussion

The study demonstrated successful implementation of a virtual UE exercise program (ie, virtual GRASP) delivered via videoconferencing in a group setting for individuals with stroke. By using the RE-AIM framework to evaluate the impact of this implementation, we demonstrated the effectiveness of the program on improving UE function and self-perceived hand function and recovery. Next steps to inform the future delivery of this virtual program were also identified. These findings are valuable to facilitate the broader implementation of this virtual program and guide the field of community-based telerehabilitation, especially during the COVID-19 pandemic, for which measures such as social distancing were imposed to limit person-to-person contacts.

The findings of this study demonstrated that the virtual GRASP program had similar effects on improving self-perceived hand function as the in-person GRASP program in our previous study. As the evaluation was conducted virtually via videoconferencing, commonly used clinical assessments (eg, Fugl-Meyer Assessment, Action Research Arm Test, grip strength) requiring special equipment (eg, a dynamometer, various sizes of blocks) or the examiner to palpate or give resistance were not used in this study. Thus, a newly developed assessment, the ArmCAM test,^15^ designed to evaluate UE function remotely through videoconferencing was used. Although not all comparisons were significant, medium to large effect sizes were found. Given the sample size is small, it is possible that the study is underpowered, which reduces the likelihood of detecting a true effect.

Similar to our previous study^11^ implementing the in-person GRASP community program at a local community center, the following essential components were also found for successful implementation of the virtual format of the GRASP program: the program structure, accessible resources, positive group dynamics, and high program fidelity. Moreover, the virtual format was more accessible geographically compared to the in-person program with the ability to *reach* individuals who would not be able to come to weekly in-person classes such as people living in remote areas and in different provinces. Attendance was higher than the in-person GRASP community program, possibly due to less commute stress. Other advantages related to videoconferencing technology may also contribute to the successful implementation, including the use of breakout rooms to allow for individualized exercises progression in smaller groups and the screen sharing to allow for sharing exercises videos/pictures efficiently.

While the videoconferencing technology enabled the GRASP program to be delivered in a virtual format, it also introduced challenges associated with technical difficulties and therefore disrupted the class content agenda. As the SRABC/MODC staff had previous experience in delivering the in-person GRASP community program, factors affecting program uptake reported by staff were primarily due to its adaptions to the virtual format. The most-reported challenge by the instructor and volunteers was the nonideal and limited webcam views for them to observe participants’ UE movements, especially when using a monitor with a built-in webcam or using a phone/tablet that requires a holder to place the device at the ideal angle. Although an egg carton holder (Suppl. Appendix 4) was able to place the phone properly, it required more time to adjust the webcam for a clear view. Therefore, future delivery of the program should familiarize the participants with their own videoconferencing setup, which should be performed prior to the first class or during the first class. Another challenge was to manage the class when multiple participants were talking simultaneously as it could be distracting, especially for people with aphasia to understand and join the conversation. Videoconferencing etiquette should be introduced in the first class and brought up again when needed. For example, be respectful and do not interrupt other people when they are speaking, physically raise your hand if you want to speak, avoid side conservations, and allow the instructor to mute participants if needed to manage background noise.

### Limitations

The study has several limitations. First, this is a study from 2 iterations in 1 setting. Additional insights may arise with more iterations or with scaling up of the programs. Second, correction for multiple comparisons was not applied because it may cause us to miss out on potential differences with the small sample size and low power. Future studies with a larger sample size and multiple comparison correction are needed to confirm our findings. Furthermore, our study excluded individuals who were not able to log into a videoconference to attend the class, which also limited the generalizability. However, we demonstrated a high level of reach to individuals with stroke in remote areas and other provinces, showing the potential of scaling up this virtual GRASP program.

## Conclusions

This study demonstrated the feasibility of implementing the virtual format of the GRASP program delivered via videoconferencing by a nonprofit organization. We also demonstrated the effectiveness of this virtual program on improving UE motor function and self-perceived hand function in a small sample of individuals with stroke. By evaluating each RE-AIM component, the study provided useful insights to inform the next steps for future iterations and scaling-up and, most important, to guide the field of telerehabilitation during the COVID-19 pandemic and beyond.

## Supplementary Material

Supplemental_Appendix_1_pzab083

Supplemental_Appendix_2_pzab083

Supplemental_Appendix_3_pzab083

Supplemental_Appendix_4_pzab083
