# Supplementary material for: Implementation and Evaluation of the Virtual Graded Repetitive Arm Supplementary Program (GRASP) for Individuals With Stroke During the COVID-19 Pandemic and Beyond
Source: Phys Ther. 2021 Mar 4;101(6):pzab083. doi: 10.1093/ptj/pzab083 (PMC7989195; doi:10.1093/ptj/pzab083)
Supplement: Supplemental_Appendix_1_pzab083 [file supplemental_appendix_1_pzab083.docx]

**Supplemental Appendix 1. The role of the research team and SRABC/MODC staff during the implementation**

| Role | Responsibilities |
| --- | --- |
| Research team as an outreach facilitator ^a^ | - Provided support and consultation to assist transforming the in-person community program to a virtual format delivered by the SRABC/MODC. |
| SRABC/MODC regional coordinator | - Provided operational and logistical oversight including in-kind promotion, administration, instructors and volunteers recruitment, and supervision of the instructor. - Set cost-recovery fees to cover the salary of the instructor. |
| SRABC/MODC virtual GRASP program instructor | - Screened potential participants - Led the virtual GRASP program - Supervised and trained the volunteers |
| SRABC/MODC virtual GRASP volunteer | - Assisted with classes under the supervision by the program instructor - Followed the instructor’s programming instructions for exercises in each class - Made autonomous decision about exercises progression and modification for the participants assigned by the instructor in the Zoom breakout rooms |

^a^ Individuals external to the organization trained to assist others to improve performance through a formal implementation process ^12^.
