# Supplementary material for: Implementation and Evaluation of the Virtual Graded Repetitive Arm Supplementary Program (GRASP) for Individuals With Stroke During the COVID-19 Pandemic and Beyond
Source: Phys Ther. 2021 Mar 4;101(6):pzab083. doi: 10.1093/ptj/pzab083 (PMC7989195; doi:10.1093/ptj/pzab083)
Supplement: Supplemental_Appendix_2_pzab083 [file supplemental_appendix_2_pzab083.docx]

**Supplemental Appendix 2: GRASP practice time log sheet**

Write the number of minutes you did GRASP exercise each day.

**Note unusual events such as flu or surgery that may have prevented GRASP practice. Also note if pain or excessive fatigue.

**Month: _________**

|  | **Mon** | **Tues** | **Wed** | **Thur** | **Fri** | **Sat** | **Sun** |
| --- | --- | --- | --- | --- | --- | --- | --- |
| GRASP minutes | ______ | ______ | ______ | ______ | ______ | ______ | ______ |
| GRASP minutes | ______ | ______ | ______ | ______ | ______ | ______ | ______ |
| GRASP minutes | ______ | ______ | ______ | ______ | ______ | ______ | ______ |
| GRASP minutes | ______ | ______ | ______ | ______ | ______ | ______ | ______ |
| GRASP minutes | ______ | ______ | ______ | ______ | ______ | ______ | ______ |
| GRASP minutes | ______ | ______ | ______ | ______ | ______ | ______ | ______ |
