# Supplementary material for: Implementation and Evaluation of the Virtual Graded Repetitive Arm Supplementary Program (GRASP) for Individuals With Stroke During the COVID-19 Pandemic and Beyond
Source: Phys Ther. 2021 Mar 4;101(6):pzab083. doi: 10.1093/ptj/pzab083 (PMC7989195; doi:10.1093/ptj/pzab083)
Supplement: Supplemental_Appendix_3_pzab083 [file supplemental_appendix_3_pzab083.docx]

**Supplemental Appendix 3**. Typical class agenda and components

| **Time** | **Agenda** | **Components** |
| --- | --- | --- |
| 15 mins | Experience sharing and discussion | The instructor led discussions on the following topics:  1) how participants used their affected arms in the previous week, 2) specific relevant GRASP exercises and activities of daily living, and 3) facilitators and barriers to doing the GRASP exercises at home.  Examples: How did the GRASP exercises go in the past week? What new exercises/ activities of daily living did you try in the past week? Did you have any difficulties while doing the GRASP exercises/ activities of daily living? |
| 35 mins | Exercises progression and modification | Exercises are progressed and modified by the instructor and volunteers if needed. Zoom breakout rooms were used to separate participants into small group. A maximum of two participants were assigned to one instructor/volunteer) in one breakout room. |
| 10 mins | Goal setting and wrap-up | The instructor reviewed weekly log sheets.  Example: Any barriers related to incomplete practice? |
|  |  | Participants completed self-assessments on how they have used their affected arm and hand in the previous week.  Example: How much did you use your affected arm and hand in your daily activities in the last 7 days (less than/ same as/ more than last week)? |
|  |  | Participants named new things that they would do with their affected arms and hands in the coming week.  Example: What new daily activity will you do to increase the use of your affected arm and hand in the coming week? |
