# Supplementary figures and images for: Implementation and Evaluation of the Virtual Graded Repetitive Arm Supplementary Program (GRASP) for Individuals With Stroke During the COVID-19 Pandemic and Beyond
Source: Phys Ther. 2021 Mar 4;101(6):pzab083. doi: 10.1093/ptj/pzab083 (PMC7989195; doi:10.1093/ptj/pzab083)

**Supplemental Appendix 4**. An egg carton to hold the device.


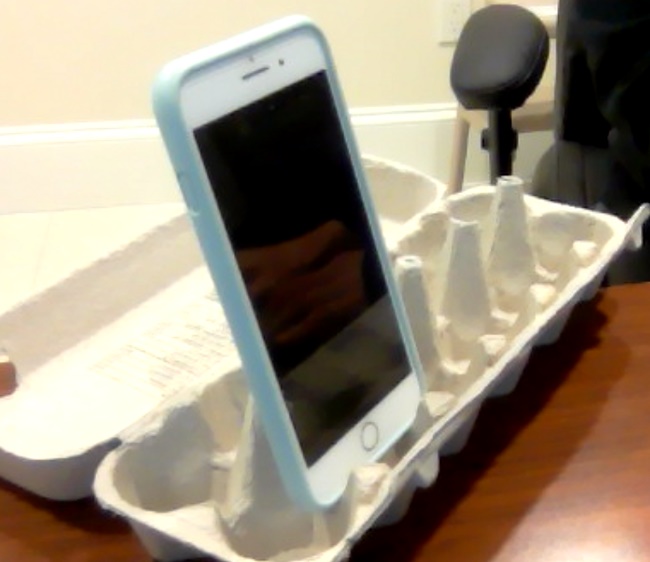

Supplement: Supplemental_Appendix_4_pzab083 [file supplemental_appendix_4_pzab083.docx]
